# Supplementary material for: Synergistic Antibiotic Activity of Ricini Semen Extract with Oxacillin against Methicillin-Resistant Staphylococcus aureus
Source: Antibiotics (Basel). 2023 Feb 6;12(2):340. doi: 10.3390/antibiotics12020340 (PMC9952580; doi:10.3390/antibiotics12020340)
Supplement: Supplementary file 1 [file antibiotics-12-00340-s001.zip › antibiotics-2189076-supplementary.pdf]

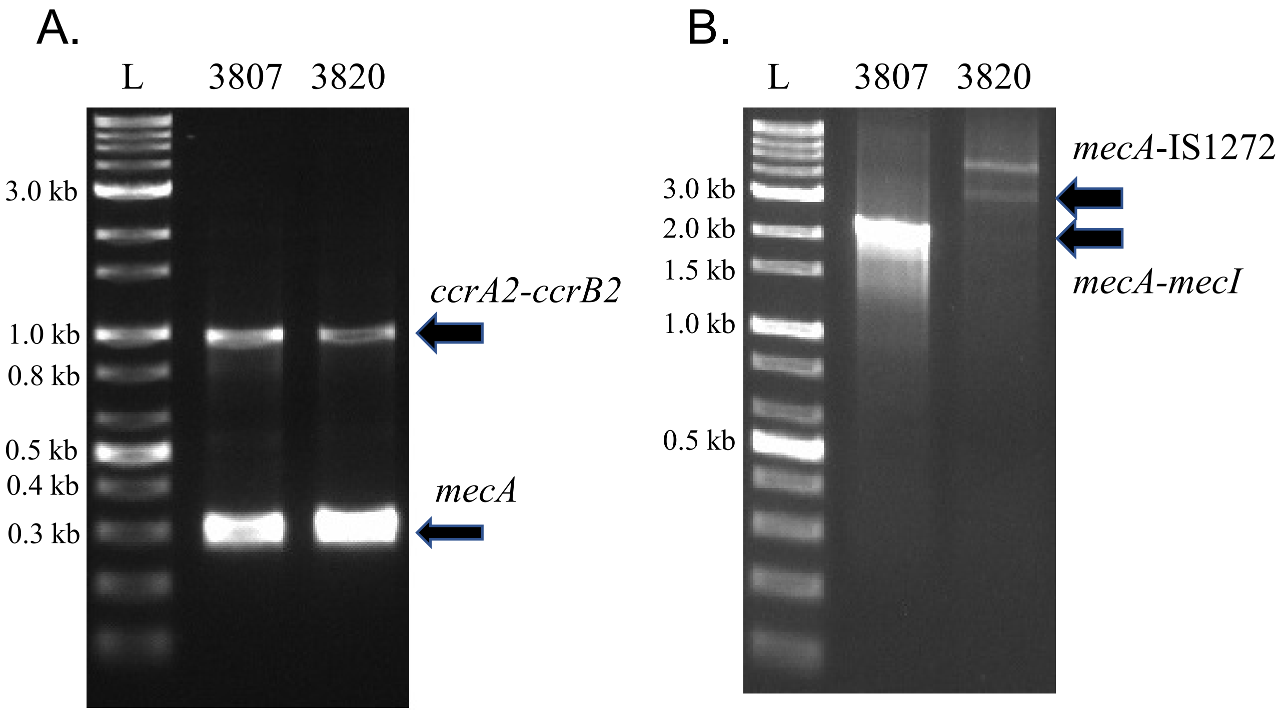

Supplementary Figure S1. SCC*mec* element type of MRSA CCARM 3807 and MRSA CCARM 3820. The presence of the SCC*mec* related genes were confirmed using PCR. The presence of *ccrA2-ccrB2* and *mecA* confirm the presence of the SCC*mec* element and are indicated by the arrows (A). Selective existence of *mecA*-IS1272 or *mecA-mecI* was checked to identify the type of SCC*mec* element. L: DNA size marker (kilobase); 3807: MRSA CCARM 3807; 3820: MRSA CCARM 3820.
